# Supplementary figures and images for: A single DNA methylation site regulates cell fate during Clostridioides difficile sporulation
Source: PLoS Pathog. 2026 Jul 23;22(7):e1013845. doi: 10.1371/journal.ppat.1013845 (PMC13395437; doi:10.1371/journal.ppat.1013845)

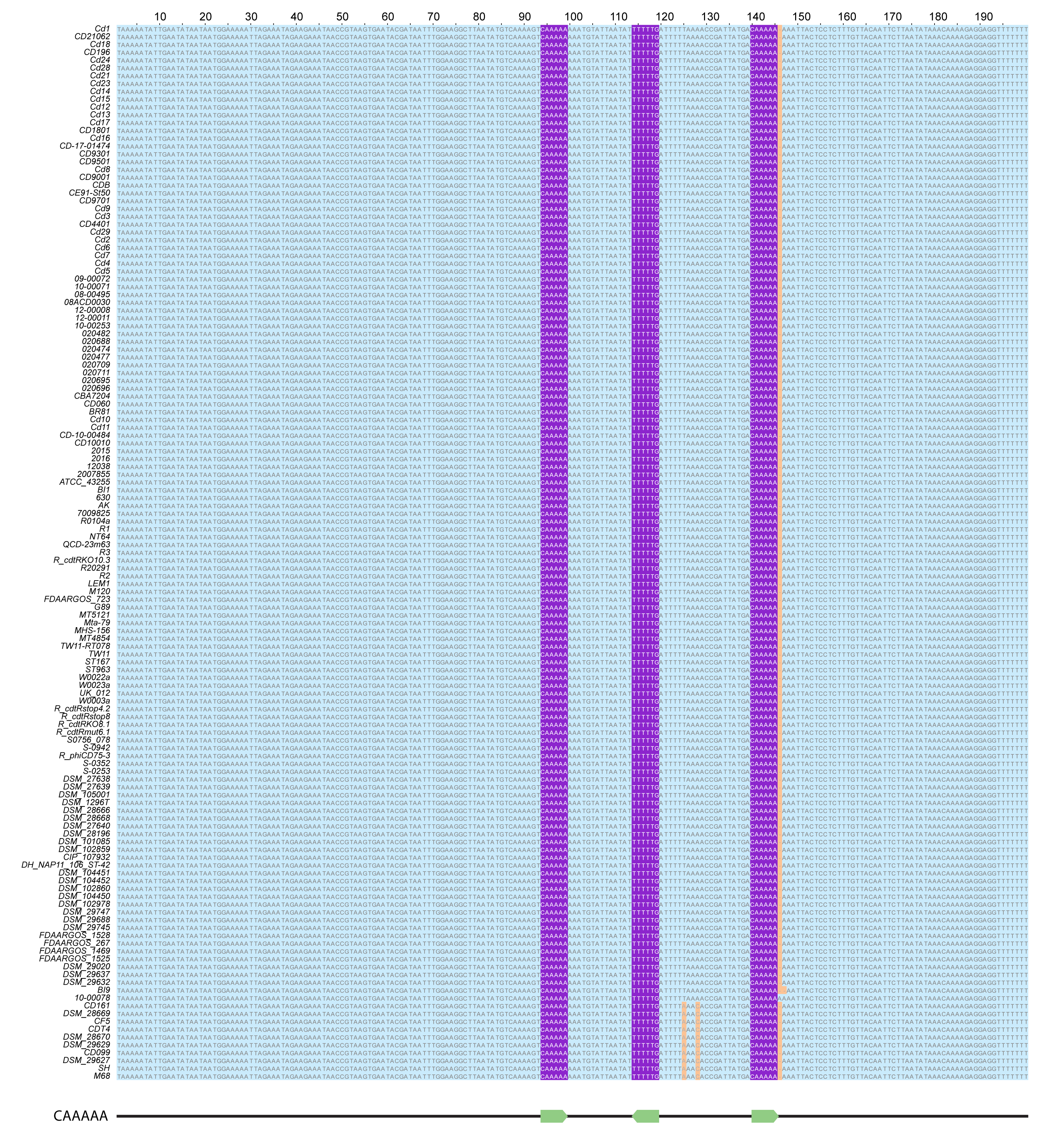

Supplement: S1 Fig — (TIF) [file ppat.1013845.s001.tif]

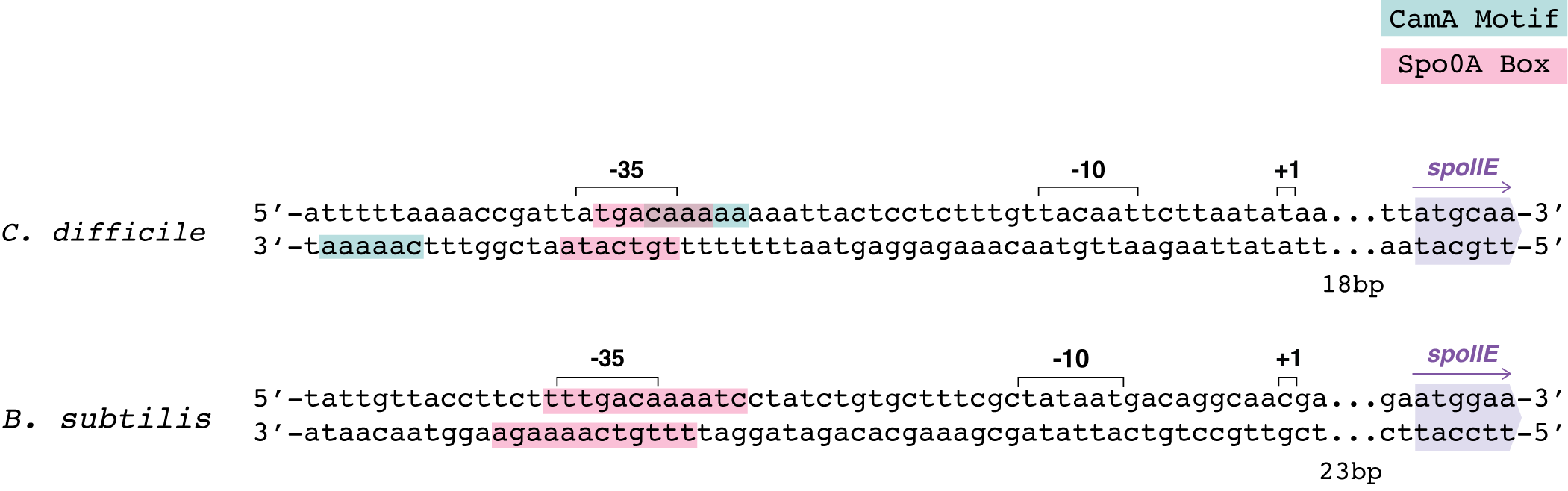

Supplement: S2 Fig — (TIF) [file ppat.1013845.s002.tif]

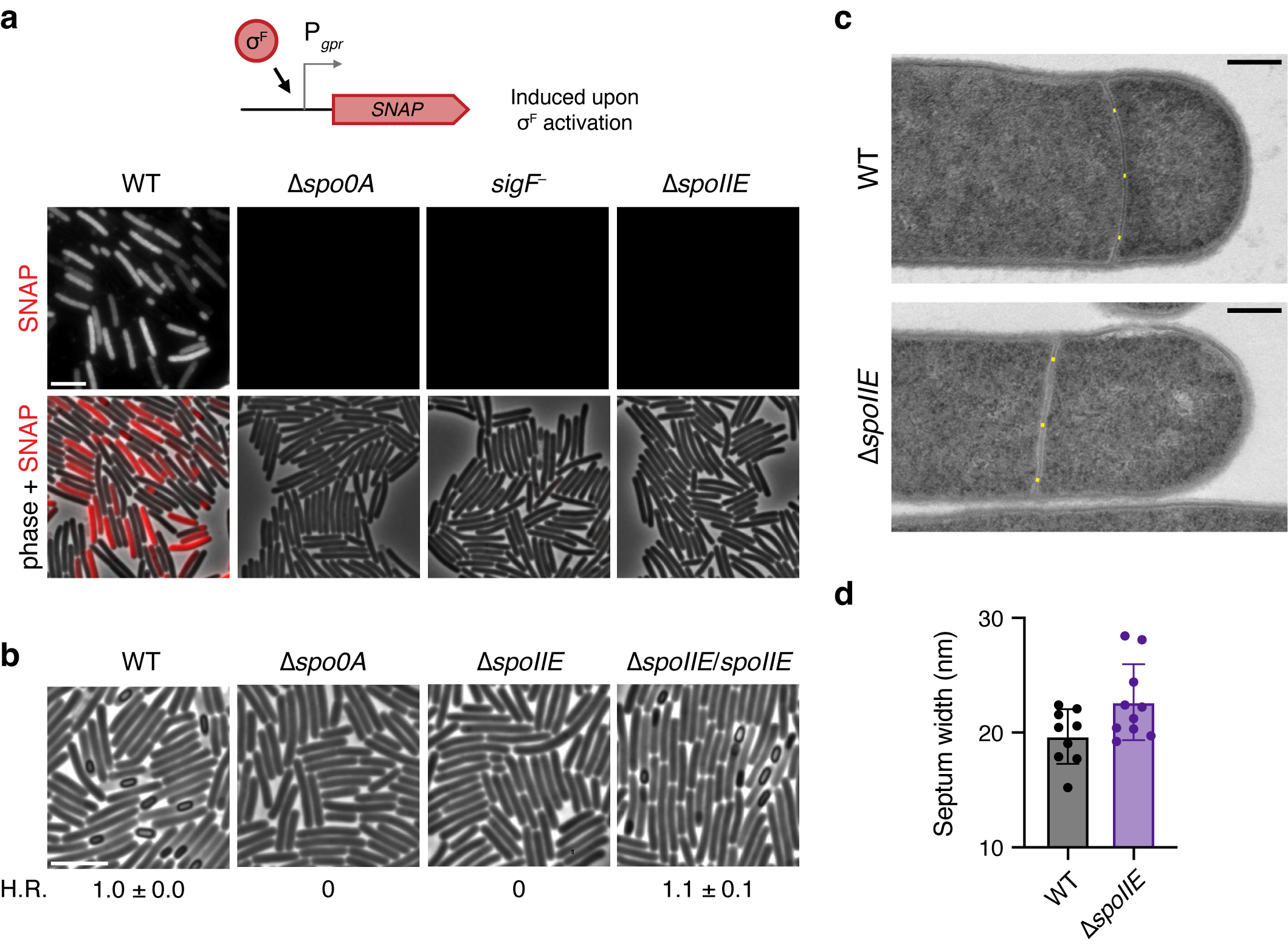

Supplement: S3 Fig — (TIF) [file ppat.1013845.s003.tif]

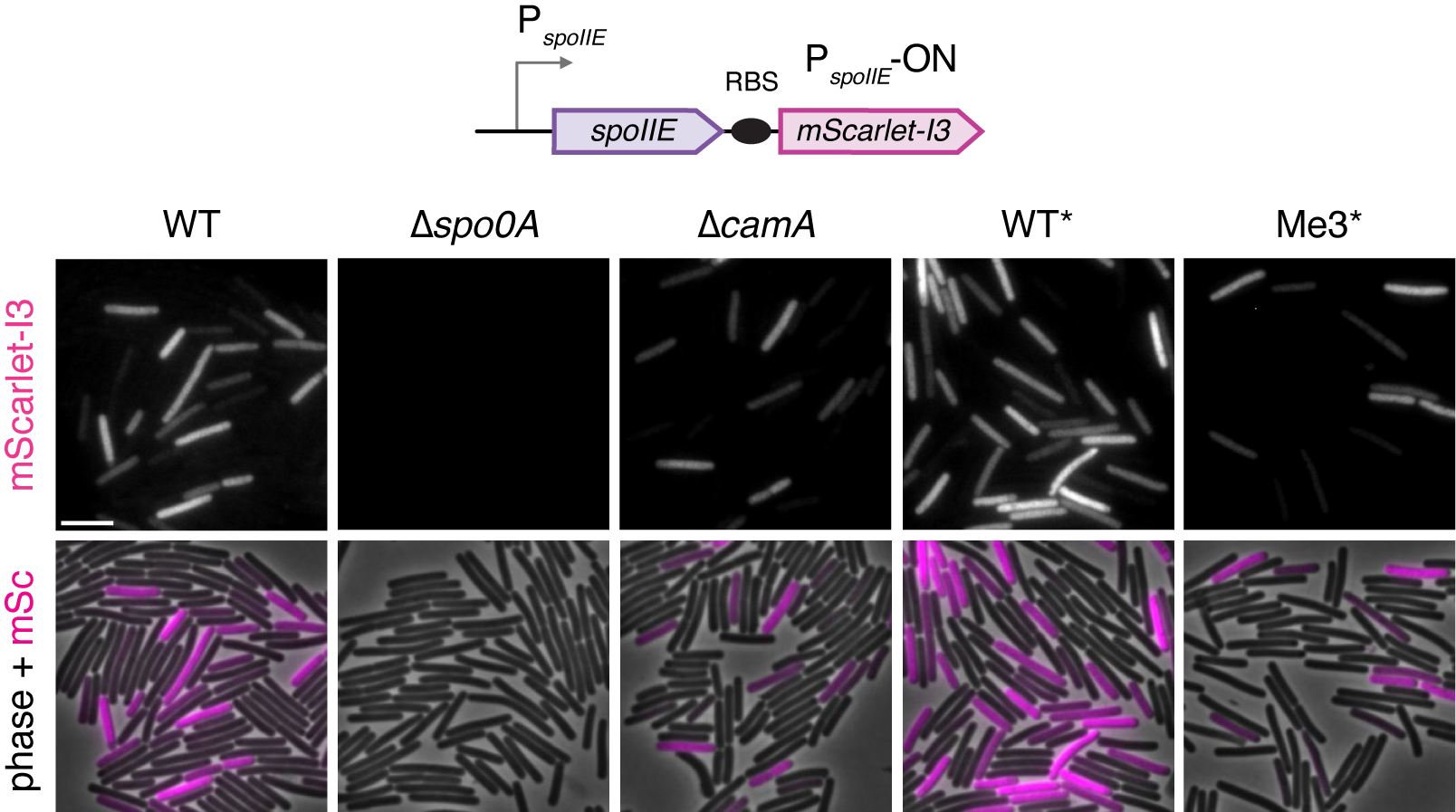

Supplement: S4 Fig — (TIF) [file ppat.1013845.s004.tif]

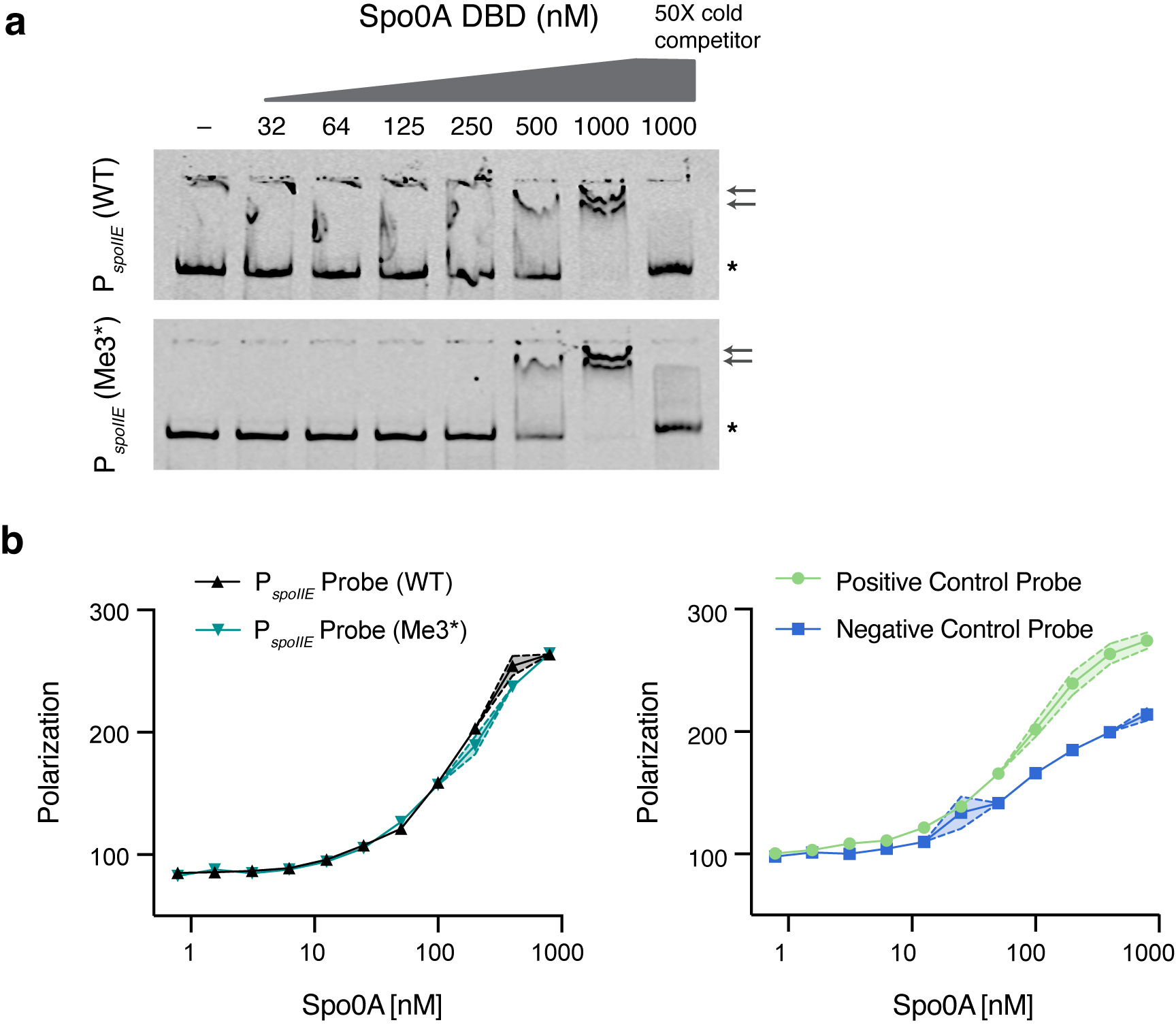

Supplement: S5 Fig — (TIF) [file ppat.1013845.s005.tif]

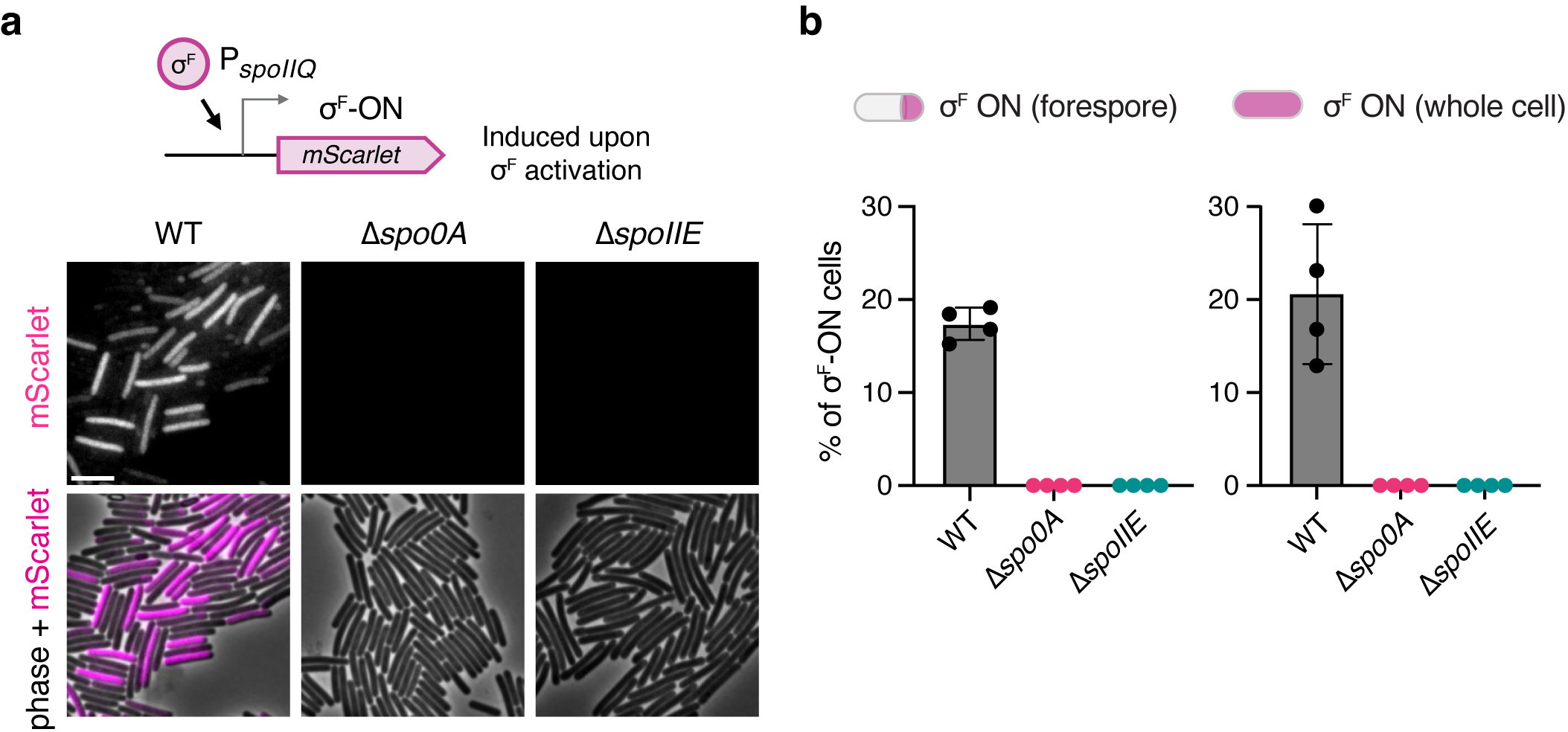

Supplement: S6 Fig — (TIF) [file ppat.1013845.s006.tif]

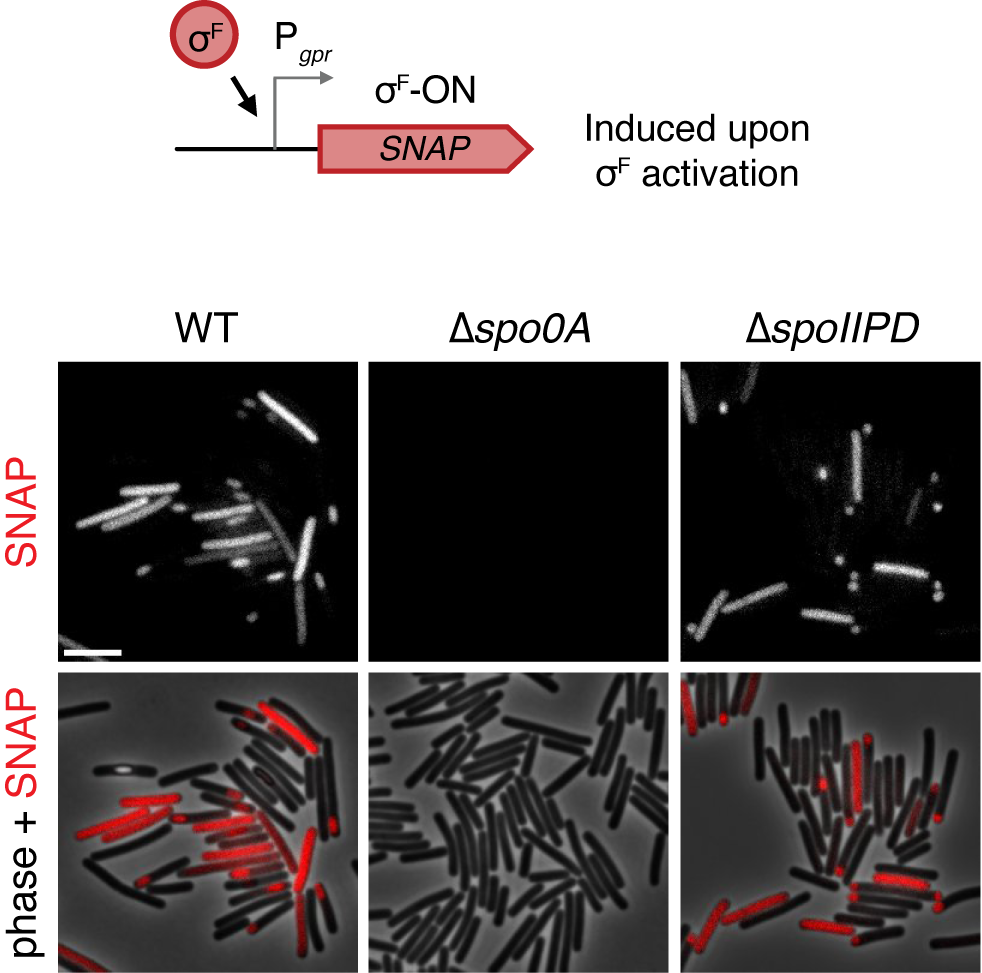

Supplement: S7 Fig — (TIF) [file ppat.1013845.s007.tif]

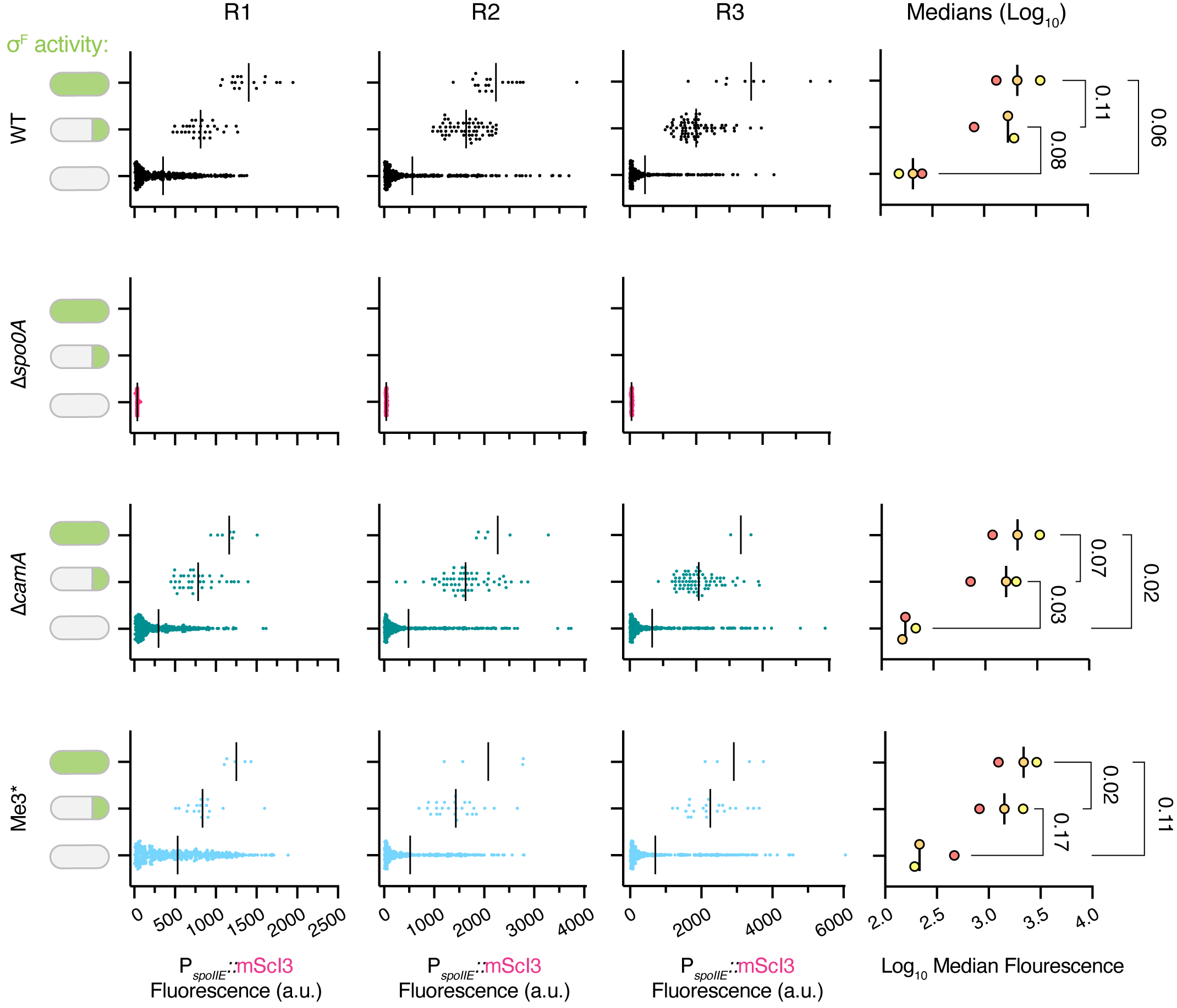

Supplement: S8 Fig — (TIF) [file ppat.1013845.s008.tif]

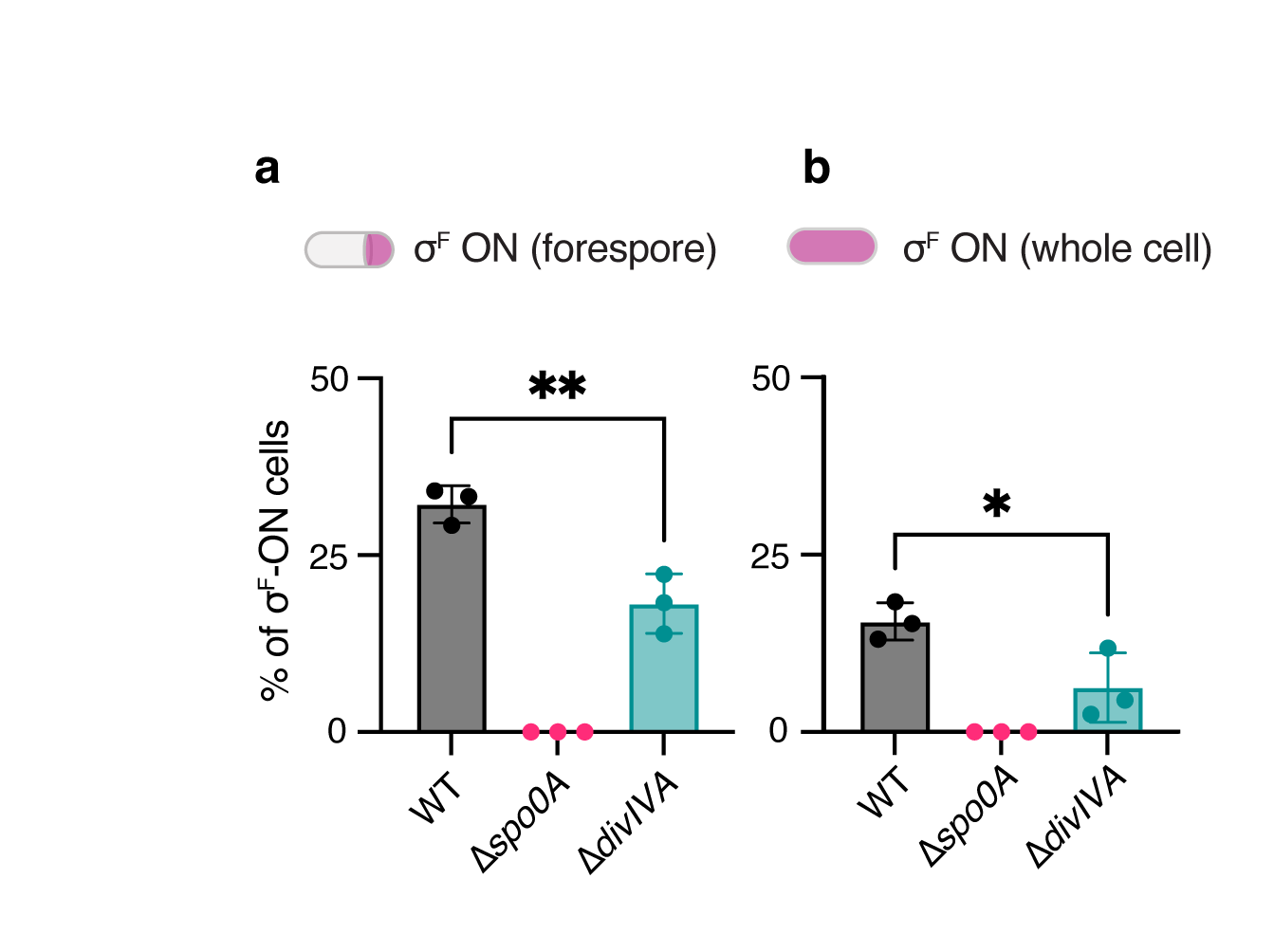

Supplement: S9 Fig — (TIF) [file ppat.1013845.s009.tif]

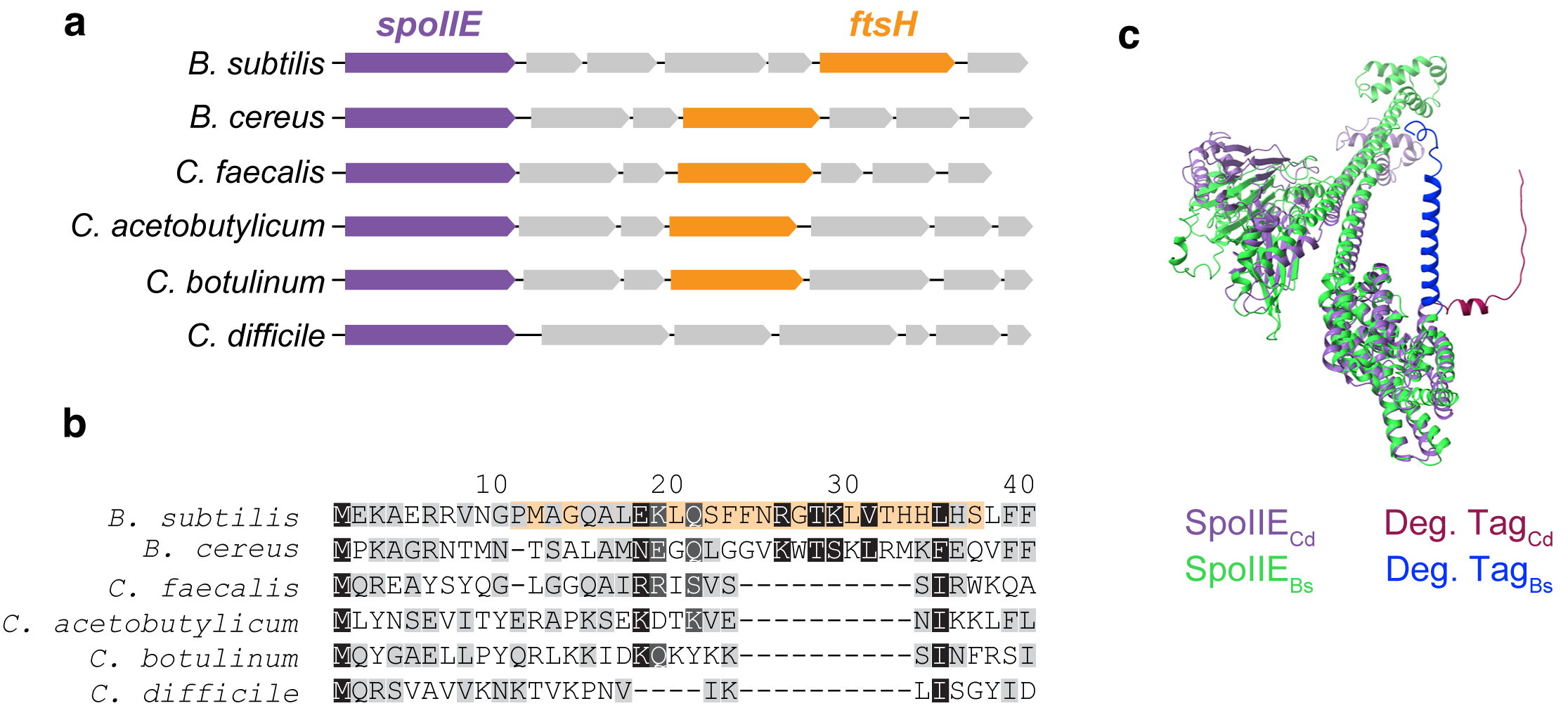

Supplement: S10 Fig — (TIF) [file ppat.1013845.s010.tif]

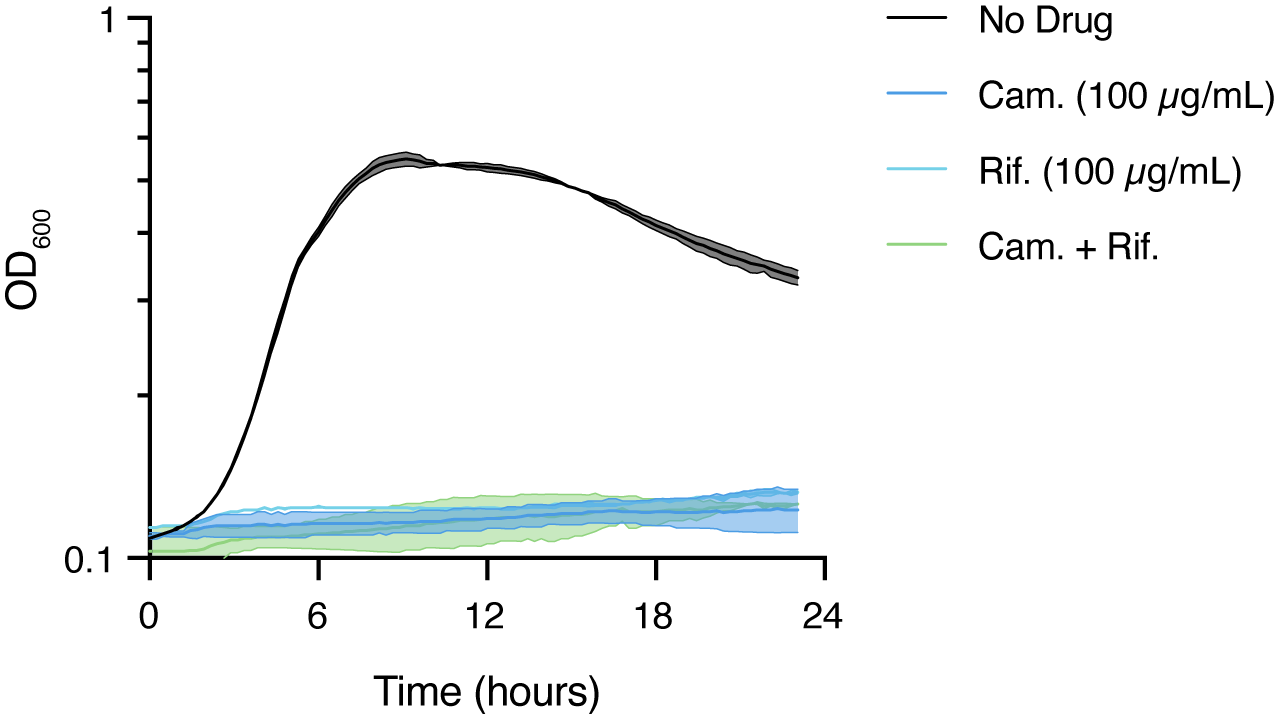

Supplement: S11 Fig — (TIF) [file ppat.1013845.s011.tif]

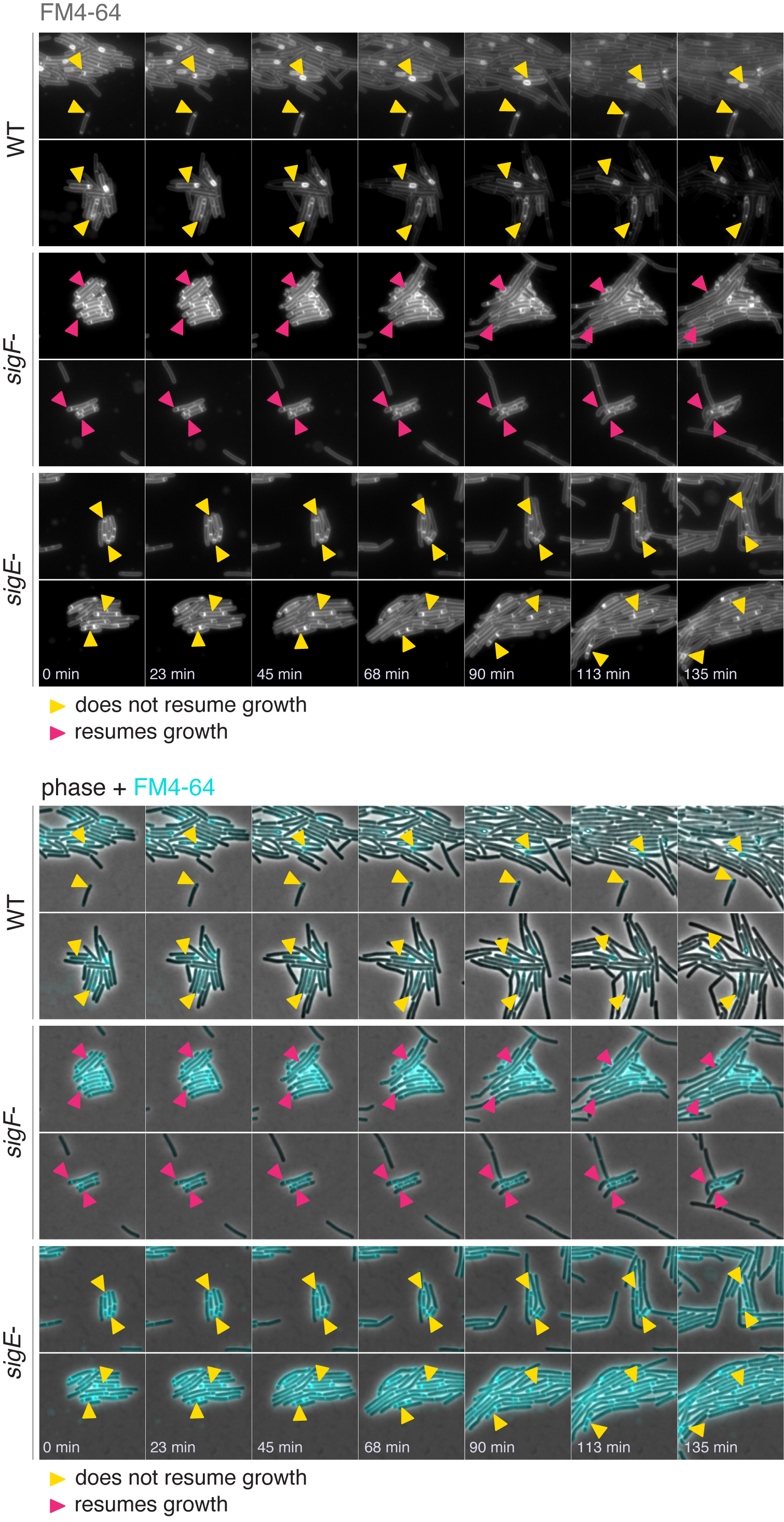

Supplement: S12 Fig — (TIF) [file ppat.1013845.s012.tif]

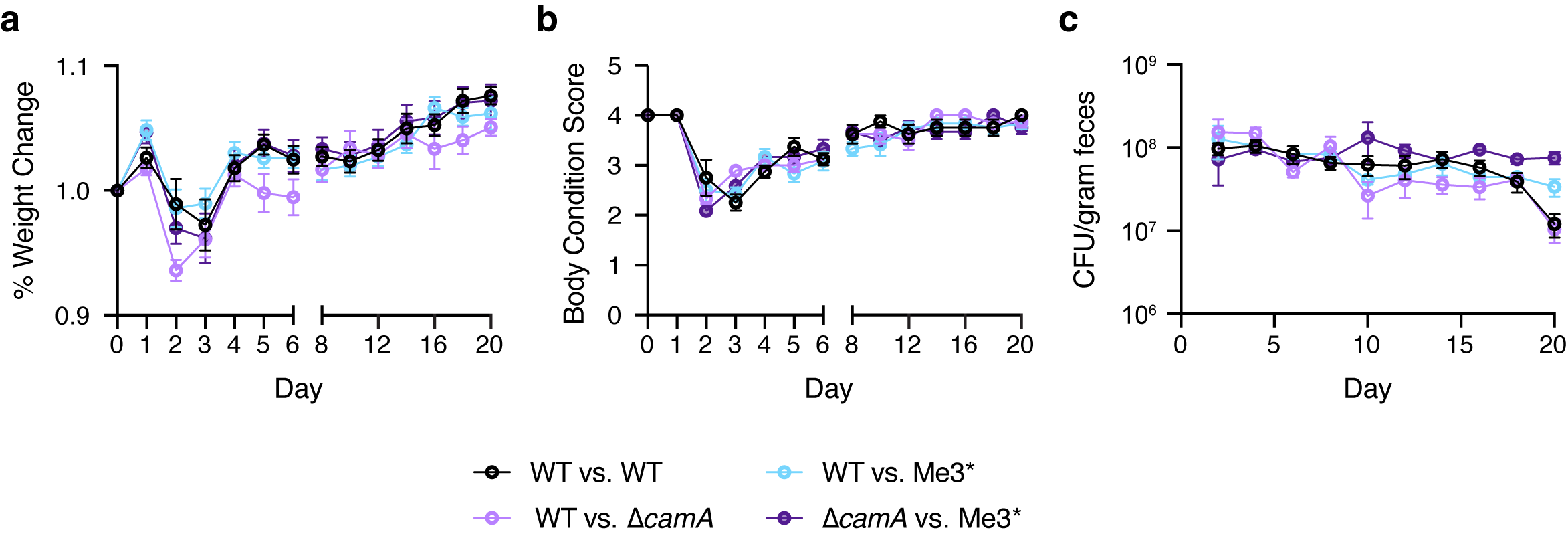

Supplement: S13 Fig — (TIF) [file ppat.1013845.s013.tif]

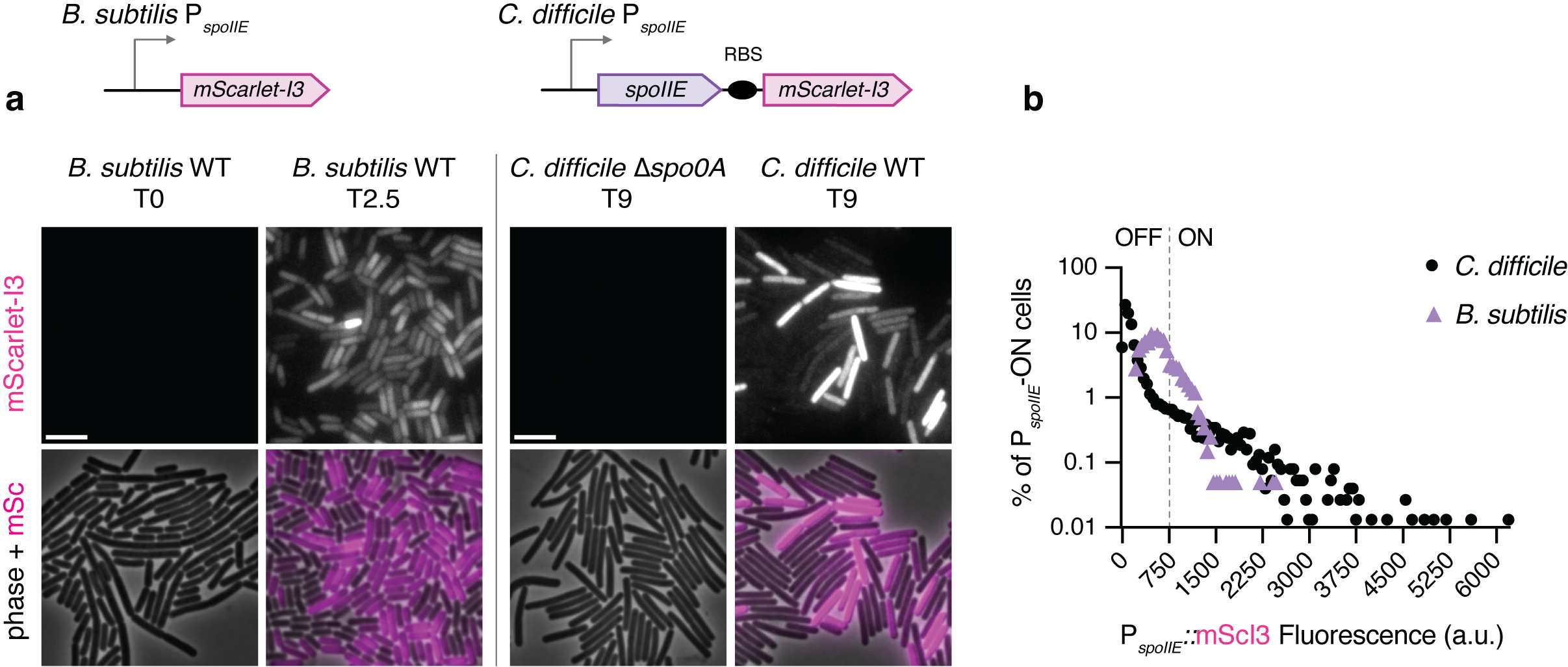

Supplement: S14 Fig — (TIF) [file ppat.1013845.s014.tif]

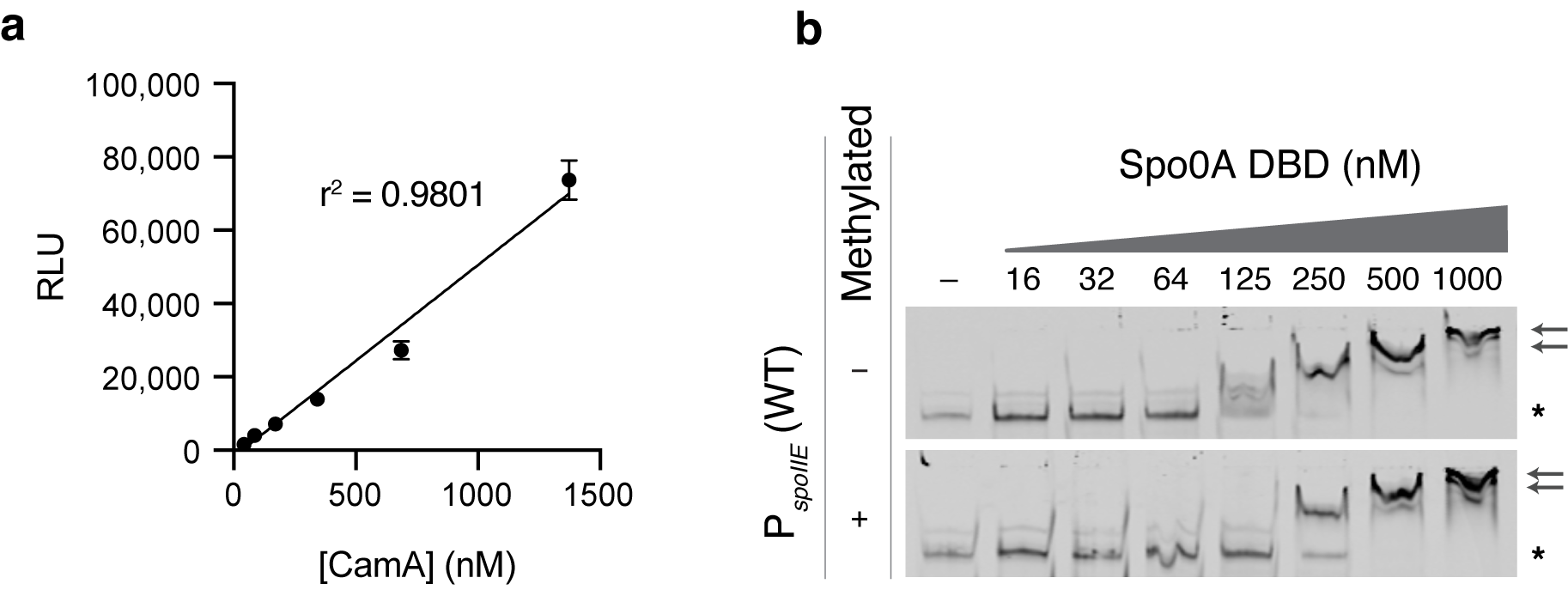

Supplement: S15 Fig — (TIF) [file ppat.1013845.s015.tif]

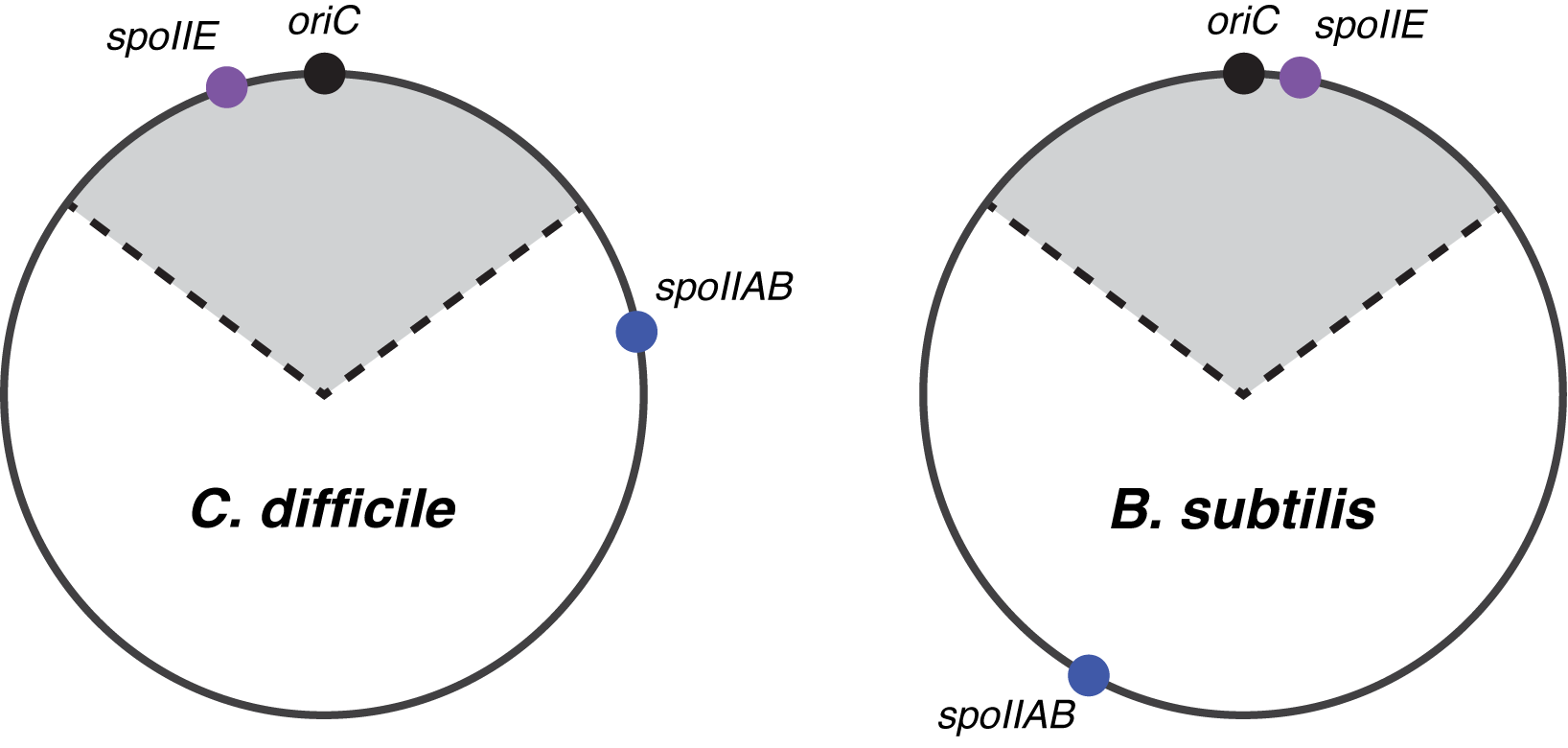

Supplement: S16 Fig — (TIF) [file ppat.1013845.s016.tif]
