# Supplementary material for: A single DNA methylation site regulates cell fate during Clostridioides difficile sporulation
Source: PLoS Pathog. 2026 Jul 23;22(7):e1013845. doi: 10.1371/journal.ppat.1013845 (PMC13395437; doi:10.1371/journal.ppat.1013845)
Supplement: S1 Table — (PDF) [file ppat.1013845.s017.pdf]

Supplementary Table 1. Genome accession numbers used in Supplem

Strain	# Assembly Accession	Genome Size
08ACD0030	GCF_001577795.1	4167076
BR81	GCF_002007885.1	4124384
FDAARGOS 267	GCF_002073735.2	4286148
DH/NAP11/106/ST-42	GCF_002234355.1	4087127
R0104a	GCF_002812585.1	4190038
W0023a	GCF_002812605.1	4110080
W0022a	GCF_002812625.1	4188456
W0003a	GCF_002812645.1	4075361
CD-10-00484	GCF_002945415.1	4119484
CD-17-01474	GCF_002945515.1	4201330
12-00008	GCF_002945665.1	4119604
08-00495	GCF_002945755.1	4177956
12-00011	GCF_002945855.1	4119932
10-00071	GCF_002945945.1	4119720
10-00078	GCF_002946035.1	4119749
10-00253	GCF_002946135.1	4120065
09-00072	GCF_002946195.1	4119929
R1	GCF_002946515.2	4093143
R3	GCF_002946535.2	4093147
R2	GCF_002946555.2	4093145
AK	GCF_002954015.1	4315250
CDT4	GCF_003095695.1	4289548
DSM 27638	GCF_003313545.1	4229698
DSM 27639	GCF_003313565.1	4263997
DSM 27640	GCF_003313585.1	4229629
DSM 102859	GCF_003456975.1	4247015
DSM 102860	GCF_003457015.1	4253785
DSM 102978	GCF_003457035.1	4253783
DSM 29688	GCF_003481905.1	4228964
DSM 29632	GCF_003481965.1	4175826
DSM 28196	GCF_003482035.1	4205365
DSM 28666	GCF_003482065.1	4122585
DSM 28669	GCF_003482125.1	4137693
DSM 29020	GCF_003482165.1	4139418
DSM 28670	GCF_003482225.1	4191653
DSM 29627	GCF_003482255.1	4209547
DSM 29629	GCF_003482305.1	4111235
DSM 29637	GCF_003482325.1	4114585
DSM 29745	GCF_003482345.1	4240646
DSM 29747	GCF_003482365.1	4071596
DSM 28668	GCF_003490105.1	4214812

20688	GCF_003597795.1	4221948
20695	GCF_003597815.1	4174373
20696	GCF_003597835.1	4119431
20709	GCF_003597855.1	4091287
20477	GCF_003597875.1	4144005
20482	GCF_003597895.1	4182371
20711	GCF_003597915.1	4174406
CD10010	GCF_003697205.1	4049580
12038	GCF_003697225.1	4077502
CD21062	GCF_003697245.1	4105481
Mta-79	GCF_008245165.1	4123685
TW11	GCF_009362915.1	4142594
FDAARGOS 723	GCF_009730495.1	4178106
CBA7204	GCF_009867095.1	4038993
DSM 105001	GCF_014236775.1	4198599
DSM 101085	GCF_015732535.1	4045635
CD9701	GCF_016766975.1	4001197
CD9501	GCF_016766995.1	4077288
CD9301	GCF_016767015.1	3955466
CD9001	GCF_016767035.1	3951345
CD4401	GCF_016767055.1	4004108
CD1801	GCF_016767095.1	4184560
7009825	GCF_016767115.1	4065486
2016	GCF_016767135.1	4070623
S0756 078	GCF_017592625.2	4071782
2015	GCF_018255775.1	4208592
MT4854	GCF_018603395.1	3998618
MHS-156	GCF_018603455.1	3998707
MT5121	GCF_018603475.1	3998712
Cd29	GCF_018884605.1	4186793
Cd28	GCF_018884625.1	4307593
Cd24	GCF_018884645.1	4071445
Cd23	GCF_018884665.1	4149746
Cd21	GCF_018884685.1	4113713
Cd18	GCF_018884705.1	4356569
Cd17	GCF_018884725.1	3936124
Cd16	GCF_018884745.1	4203966
Cd14	GCF_018884765.1	4240901
Cd15	GCF_018884785.1	4393991
Cd13	GCF_018884805.1	4102642
Cd12	GCF_018884825.1	4100358
Cd11	GCF_018884845.1	4404202
Cd10	GCF_018884865.1	4245736
Cd9	GCF_018884885.1	4121924
Cd8	GCF_018884905.1	4274902

Cd7	GCF_018884925.1	4292701
Cd5	GCF_018884945.1	4272298
Cd6	GCF_018884965.1	4321901
Cd4	GCF_018884985.1	4184598
Cd3	GCF_018885005.1	4641978
Cd2	GCF_018885025.1	4261386
Cd1	GCF_018885045.1	4282853
S-0352	GCF_018885065.1	4251987
S-0253	GCF_018885085.1	4095894
S-0942	GCF_018885105.1	4129159
FDAARGOS 1469	GCF_019931025.1	4045616
FDAARGOS 1528	GCF_020097215.1	4375351
FDAARGOS 1525	GCF_020341515.1	4307330
CDB	GCF_022695655.1	4234172
CE91-St50	GCF_022845595.1	4293712
DSM 104452	GCF_024399435.1	4167361
NT64	GCF_024496065.1	4078891
UK 012	GCF_024628745.1	4535071
ST963	GCF_024628765.1	4476372
SH	GCF_024972115.1	4287439
CD099	GCF_024972275.1	4302821
G89	GCF_024972415.1	4288937
CD060	GCF_024972555.1	4338261
DSM 104450	GCF_025135955.1	4270750
DSM 104451	GCF_025136135.1	4164224
R cdtRstop8	GCF_025758145.1	4204879
R cdtRstop4.2	GCF_025758165.1	4204750
R cdtRmut6.1	GCF_025758185.1	4204869
R phiCD75-3	GCF_025758205.1	4233065
R cdtRKO8.1	GCF_025758225.1	4204192
R cdtRKO10.3	GCF_025758245.1	4204257
QCD-23m63	GCF_000155065.1	3940285
CIP 107932	GCF_000155045.1	4036680
2007855	GCF_000210455.1	4179867
BI1	GCF_000211235.1	4464700
CF5	GCF_000210415.1	4159517
M68	GCF_000210395.1	4308325
DSM 1296T	GCF_001077535.1	4154879
20474	GCF_003597775.1	4108203
BI9	GCF_000270485.1	4178227
LEM1	GCF_001984465.1	4191506
TW11-RT078	GCF_011045515.1	3592931
630	GCF_000932055.2	4274782
ATCC 43255	GCF_000155025.1	4206980
CD161	GCF_003095675.1	4474333

CD196	GCF_021378415.1	4121608
M120	GCF_000210435.1	4047729
R20291	GCF_015732555.1	4204902
ST167	GCF_024919155.1	3949751

ental Figure 1

GC percent
29.0
28.5
28.5
28.5
28.5
28.5
29.0
28.5
28.5
29.0
28.5
28.5
28.5
28.5
28.5
28.5
28.5
29.0
29.0
29.0
28.5
29.0
29.0
29.0
29.0
29.0
29.0
29.0
29.0
28.5
29.0
29.0
29.0
29.0
29.0
29.0
28.5
28.5
29.0
29.0
28.5

29.0
28.5
29.0
28.5
29.0
29.0
28.5
28.5
29.0
29.0
28.5
29.0
28.5
28.5
29.0
28.5
28.5
28.5
28.5
28.5
28.5
29.0
29.0
29.0
29.0
29.0
28.5
28.5
28.5
28.5
28.5
28.5
28.5
28.5
28.5
28.5
28.5
29.0
28.5
28.5
28.5
28.5
28.5
28.5
28.5

28.5
28.5
29.5
29.0
28.5
28.5
28.5
29.0
28.5
28.5
28.5
29.0
28.5
29.0
29.0
29.0
28.5
29.5
28.5
29.0
29.0
29.0
29.0
29.0
29.0
29.0
29.0
29.0
29.0
29.0
29.0
28.5
28.5
28.5
28.5
28.5
29.0
29.0
29.0
28.5
29.0
29.5
29.0
28.5
29.0

28.5
28.5
29.0
28.5
