# Supplementary material for: A single DNA methylation site regulates cell fate during Clostridioides difficile sporulation
Source: PLoS Pathog. 2026 Jul 23;22(7):e1013845. doi: 10.1371/journal.ppat.1013845 (PMC13395437; doi:10.1371/journal.ppat.1013845)
Supplement: S2 Table — (PDF) [file ppat.1013845.s018.pdf]

Supplementary Table 2. *C. difficile* strains used in this study

Lab Strain #	Strain name	Relevant genotype
756	630Δ*erm* Δ*pyrE*	*erm*-sensitive derivate of 630 with a deletion in *pyrE*
846	630Δ*erm* -*pyrE*	*erm*-sensitive derivate of 630 with *pyrE* restored
787	630Δ*erm* Δ*pyrE* Δ*spo0A*	630ΔermΔpyrE with *spo0A* deleted
849	630Δ*erm* -*pyrE* Δ*spo0A*	630Δ*erm* Δ*spo0A* with *pyrE* restored
1402	630Δ*erm* Δ*pyrE* Δ*camA*	630Δ*erm* Δ*pyrE* with *camA* deleted
1526	630Δ*erm* -*pyrE* Δ*camA*	630Δ*erm* Δ*camA* with *pyrE* restored
5103	630Δ*erm* Δ*pyrE* / *spoIIE*-RBS-*mScarlet-I3*	630ΔermΔpyrE with bicistronic *spoIIE-RBS-mScarlet-I3* in *spoIIE* locus
5190	630Δ*erm* -*pyrE* / *spoIIE*-RBS-*mScarlet-I3*	630Δerm with pyrE restored and bicistronic *spoIIE-RBS-mScarlet-I3* in *spoIIE* locus
5106	630Δ*erm* Δ*pyrE* Δ*spo0A* / *spoIIE*-RBS-*mScarlet-I3*	630ΔermΔpyrEΔ*spo0A* with bicistronic *spoIIE-RBS-mScarlet-I3* in *spoIIE* locus
5192	630Δ*erm* -*pyrE* Δspo0A / *spoIIE*-RBS-*mScarlet-I3*	630ΔermΔ*spo0A* with *pyrE* restored and bicistronic *spoIIE-RBS-mScarlet-I3* in *spoIIE* locus
5052	*630Δerm ΔpyrE ΔcamA* / *spoIIE*-RBS-*mScarlet-I3*	630ΔermΔpyrEΔ*camA* with bicistronic *spoIIE-RBS-mScarlet-I3* in *spoIIE* locus
5194	630Δ*erm* -*pyrE* Δ*camA* / *spoIIE*-RBS-*mScarlet-I3*	630ΔermΔ*camA* with *pyrE* restored and bicistronic *spoIIE-RBS-mScarlet-I3* in *spoIIE* locus
3830	630Δerm Δ*pyrE* Δ*IG spoIIE*	630Δ*erm*Δ*pyrE* with *spoIIE* and its upstream region deleted
4051	630Δ*erm* Δ*pyrE* WT*	630Δ*erm*Δ*pyrE*Δ*IG spoIIE w*ith the WT *spoIIE* upstream region, containing a watermark, restored in the native locus
4068	630Δ*erm* -*pyrE* WT*	630Δ*erm* WT* with *pyrE* restored
4055	630Δ*erm* Δ*pyrE* Me1*	630Δ*erm*Δ*pyrE* Δ*IG spoIIE* with the *spoIIE* upstream region, containing a CAAAAA > CAAATA mutation at Me1 and a watermark, restored in the native locus
4071	630Δ*erm* -*pyrE* Me1*	630Δ*erm* Me1* with *pyrE* restored
4059	630Δ*erm* Δ*pyrE* Me2*	630Δ*erm*Δ*pyrE* Δ*IG spoIIE* with the *spoIIE* upstream region, containing a CAAAAA > CAAATA mutation at Me2 and a watermark, restored in the native locus
4074	630Δ*erm* -*pyrE* Me2*	630Δ*erm* Me2* with *pyrE* restored
4063	630Δ*erm* Δ*pyrE* Me3*	630Δ*erm*Δ*pyrE* Δ*IG spoIIE* with the *spoIIE* upstream region, containing a CAAAAA > CAAATA mutation at Me3 and a watermark, restored in the native locus
4077	630Δ*erm* -*pyrE* Me3*	630Δ*erm* Me3* with *pyrE* restored
4028	630Δ*erm* Δ*pyrE* Δ*spoIIE*	630Δ*erm*Δ*pyrE* with the *spoIIE* deleted
4039	630Δ*erm* -*pyrE* Δ*spoIIE*	630Δ*erm* Δ*spoIIE* with *pyrE* restored
5111	630Δ*erm* Δ*pyrE* WT* / *spoIIE*-RBS-*mScarlet-I3*	630Δ*erm*Δ*pyrE* WT* with bicistronic *spoIIE-RBS-mScarlet-I3* in native locus
5196	630Δ*erm* -*pyrE* WT* / *spoIIE*-RBS-*mScarlet-I3*	630Δ*erm* WT* *spoIIE-RBS-mScarlet-I3* with *pyrE* restored
5112	630Δ*erm* Δ*pyrE* Me3* / *spoIIE*-RBS-*mScarlet-I3*	630Δ*erm*Δ*pyrE* Me3* with bicistronic *spoIIE-RBS-mScarlet-I3* in native locus
5198	630Δ*erm* -*pyrE* Me3* / *spoIIE*-RBS-*mScarlet-I3*	630Δ*erm* Me3* *spoIIE-RBS-mScarlet-I3* with *pyrE* restored
4635	630Δ*erm* -*pyrE* / P*gpr::SNAP*	630Δ*erm* with *pyrE* restored and P*gpr::SNAP* in the pyrE locus
4638	630Δ*erm* -*pyrE* Δ*spo0A* / P*gpr::SNAP*	630Δ*erm*Δ*spo0A* with *pyrE* restored and P*gpr::SNAP* in the pyrE locus
5049	630Δ*erm* - pyrE Δ*camA* / P*gpr::SNAP*	630Δ*erm*Δ*camA* with *pyrE* restored and P*gpr::SNAP* in the pyrE locus
5399	630Δ*erm* -*pyrE* Me3* / P*gpr::SNAP*	630Δ*erm* Me3* with *pyrE* restored and P*gpr::SNAP* in the pyrE locus
5156	630Δ*erm* -*pyrE* / P*gpr::SNAP* / *spoIIE*-RBS-*mScarlet-I3*	630Δ*erm* with *pyrE* restored, P*gpr::SNAP* in the *pyrE* locus and bicistronic *spoIIE-RBS-mScarlet-I3* in *spoIIE* locus
5158	630Δ*erm* -*pyrE* Δ*spo0A* / P*gpr::SNAP* / *spoIIE*-RBS-*mScarlet-I3*	630Δ*erm*Δ*spo0A* with *pyrE* restored, P*gpr::SNAP* in the *pyrE* locus and bicistronic *spoIIE-RBS-mScarlet-I3* in *spoIIE* locus
5160	630Δ*erm* -*pyrE* Δ*camA* / P*gpr::SNAP* / *spoIIE*-RBS-*mScarlet-I3*	630Δ*erm*Δ*camA* with *pyrE* restored, P*gpr::SNAP* in the *pyrE* locus and bicistronic *spoIIE-RBS-mScarlet-I3* in *spoIIE* locus
6164	630Δ*erm* -*pyrE* Me3* / P*gpr::SNAP* / *spoIIE*-RBS-*mScarlet-I3*	630Δ*erm* Me3* with *pyrE* restored, P*gpr::SNAP* in the *pyrE* locus and bicistronic *spoIIE-RBS-mScarlet-I3* in *spoIIE* locus
4721	630Δ*erm* Δ*pyrE* Δ*divIVA*	630Δ*erm*Δ*pyrE* with *divIVA* deleted
5505	630Δ*erm* -*pyrE* Δ*divIVA*	630Δ*erm*Δ*divIVA* with *pyrE* restored
5869	630Δ*erm* -*pyrE* Δ*divIVA* -*divIVA*	630Δ*erm*Δ*divIVA* with *pyrE* restored and *divIVA* complemented in the *pyrE* locus under the *cwp2* promoter
5449	630Δerm -*pyrE* Δ*divIVA* / P*gpr::SNAP*	630Δ*erm*Δ*divIVA* with *pyrE* restored and P*gpr::SNAP* in the *pyrE* locus
4862	630Δerm Δ*pyrE* / *sipL*-RBS-*mScarlet-I3*	630Δ*erm*Δ*pyrE* with bicistronic *sipL-RBS-mScarlet-I3* in the *sipL* locus
5612	630Δ*erm* -*pyrE* / *sipL*-RBS-*mScarlet-I3* / P*gpr::SNAP*	630Δ*erm* with *pyrE* restored, P*gpr::SNAP* in the *pyrE* locus and bicistronic *sipL-RBS-mScarlet-I3* in the native locus
4912	630Δ*erm* -*pyrE* -*aad9*	630Δ*erm* with *pyrE* restored and *aad9* (spectinomycin resistance gene) in the *pyrE* locus
4914	630Δ*erm* -*pyrE* Δ*camA* -*aad9*	630Δ*erm*Δ*camA* with *pyrE* restored and *aad9* (spectinomycin resistance gene) in the *pyrE* locus
5025	630Δ*erm* -*pyrE* Me3* -*aad9*	630Δ*erm* Me3* with *pyrE* restored and *aad9* (spectinomycin resistance gene) in the *pyrE* locus
1488	630Δ*erm* Δ*pyrE* *sigF*$^{-}$	630Δerm*ΔpyrE* *sigF::ermB* targetron mutant
4641	630Δ*erm* -*pyrE* *sigF*$^{-}$ / P*gpr::SNAP*	630Δ*erm* *sigF::ermB* targetron mutant with *pyrE* restored and P*gpr::SNAP* in the *pyrE* locus
4644	630Δ*erm* -*pyrE* Δ*spoIIE* / P*gpr::SNAP*	630Δ*erm*Δ*spoIIE* with *pyrE* restored and P*gpr::SNAP* in the *pyrE* locus
3856	630Δ*erm* -*pyrE* Δ*IG spoIIE* -*spoIIE*	630Δ*erm*Δ*IG spoIIE* with *pyrE* restored and *spoIIE* complemented in the *pyrE* locus
4414	630Δ*erm* -*pyrE* / P*spoIIQ::mScarlet-I3*	630Δ*erm* with *pyrE* restored and P*spoIIQ::mScarlet-I3* in the *pyrE* locus
4544	630Δ*erm* -*pyrE* Δ*spo0A* / P*spoIIQ::mScarlet-I3*	630Δ*erm*Δ*spo0A* with *pyrE* restored and P*spoIIQ::mScarlet-I3* in the *pyrE* locus
4547	630Δ*erm* -*pyrE* *sigF*$^{-}$ / P*spoIIQ::mScarlet-I3*	630Δ*erm* *sigF*- with *pyrE* restored and P*spoIIQ::mScarlet-I3* in the *pyrE* locus
1541	630Δ*erm* -*pyrE* *sigF*$^{-}$	630Δerm *sigF::ermB* targetron mutant and *pyrE* restored
1545	630Δ*erm* -*pyrE* *sigE*$^{-}$	630Δerm *sigE::ermB* targetron mutant and *pyrE* restored

1. Ng, Y.K. *et al.* Expanding the repertoire of gene tools for precise manipulation of the *Clostridium difficile* genome: allelic exchange using pyrE alleles. *PLoS One* **8**, e56051 (2013).
2. Donnelly, M.L. *et al.* A *Clostridium difficile*-Specific, Gel-Forming Protein Required for Optimal Spore Germination. *MBio* **8** (2017).
3. Oliveira, P. H. et al. Epigenomic characterization of Clostridioides difficile finds a conserved DNA methyltransferase that mediates sporulation and pathogenesis. Nat Microbiol 5, 166–180 (2020).
4. DiBenedetto, N. V., Donnelly-Morell, M. L., Kumamoto, C. A. & Shen, A. In situ visualization of Clostridioides difficile phenotypic heterogeneity and single-cell morphology during gut infection. Nat Commun 17, 1716 (2026).
5. Ribis, J. W., Fimlaid, K. A. & Shen, A. Differential requirements for conserved peptidoglycan remodeling enzymes during Clostridioides difficile spore formation. Molecular Microbiology 110, 370–389 (2018).
