# Supplementary material for: A single DNA methylation site regulates cell fate during Clostridioides difficile sporulation
Source: PLoS Pathog. 2026 Jul 23;22(7):e1013845. doi: 10.1371/journal.ppat.1013845 (PMC13395437; doi:10.1371/journal.ppat.1013845)
Supplement: S3 Table — (PDF) [file ppat.1013845.s019.pdf]

Supplementary Table 3. *E. coli* strains used in this study.

Lab Strain #	Strain Name	Relevant genotype or link to Benchling plasmid map with primers	Source/reference
41	DH5α	F^- Φ80*lacZ*ΔM15 Δ(*lacZYA-argF*) U169 *recA1 endA1 hsdR17* (rK^-, mK^+) *phoA supE44* λ– *thi-1 gyrA96 relA1*	D. Cameron
531	HB101	F^- *mcrB mrr hsdS20* (rB^-mB^-) *recA13 leuB6 ara-13 proA2 lavYI galK2 xyl-6 mtl-1 rpsL20* carrying pRK24	C. Ellermeier
-	BL21 (DE3)	fhuA2 [lon] ompT gal (λ DE3) [dcm] ∆hsdS λ DE3 = λ sBamHIo ∆EcoRI-B int::(lacI::PlacUV5::T7 gene1) i21 ∆nin5	NEB
1539	DH5α / pMTL-YN3	pMTL-YN3 in DH5α	[1]
1662	HB101 / pMTL-YN1C	pMTL-YN1C in HB101	[1]
4290	HB101 / pMTL-YN3 *spoIIE-stop*-RBS-*mScarlet-I3*	https://benchling.com/s/seq-k2VTSRw0MWS4gyh2ITvr?m=slm-Jtr8RgTke5dbE65nqSaB	This study
3145	HB101 / pMTL-YN3 Δ*IG spoIIE*	https://benchling.com/s/seq-59eTdjclsugLT4K0AsHc?m=slm-Rx9uui1JmrFYGohNJSqq	This study
3310	HB101 / pMTL-YN3 WT* *IG spoIIE* fragment	https://benchling.com/s/seq-PlhxyVsuZPaau2qBSkEM?m=slm-JdTbpKuldsvkFrUifjDA	This study
3311	HB101 / pMTL-YN3 Me1* *IG spoIIE* fragment	https://benchling.com/s/seq-euSlWm76OlUMQ9eNOG80?m=slm-NFjiV7pxl7DTlzeDwwtH	This study
3312	HB101 / pMTL-YN3 Me2* *IG spoIIE* fragment	https://benchling.com/s/seq-gcvibMQOGQt87UXfeRyj?m=slm-xFcHnKsthmYuu4dt5ODA	This study
3313	HB101 / pMTL-YN3 Me3* *IG spoIIE* fragment	https://benchling.com/s/seq-Y2qlQ6zaJRTlQF4x0cYV?m=slm-Ian4sHmPpxteihzy4c8L	This study
3276	HB101 / pMTL-YN3 ∆*spoIIE*	https://benchling.com/s/seq-eREIKlxGvE5IQI9Up31A?m=slm-UCDKDUK9xsA2PFGUHiGy	This study
3860	HB101 / pMTL-YN1C P*gpr::SNAP*	https://benchling.com/s/seq-1mmyJN4sXOKWHTZ33KIL?m=slm-oA6WuLCRVH2ugmIZdUAU	This study, adapted from [3]
3881	HB101 / pMTL-YN3 ∆*divIVA*	https://benchling.com/s/seq-xPof5aq752RBsHuaa2Ay?m=slm-5VppkOwg7egUHvhLuBQ9	This study
4865	DH5α / pMTL-YN1C P*cwp2-divIVA*	https://benchling.com/s/seq-kNGxpOWZ5ZksJq8GaYDc?m=slm-JNs97ewWW7Mt0wtjCNJ9	This study
4070	HB101 / pMTL-YN1C *aad9*	https://benchling.com/s/seq-22eERdWEhJMbGQFKwugs?m=slm-EgiS498sUu7V7BuZUMYl	This study
3688	HB101 / P*spoIIQ::mScarlet-I3*	https://benchling.com/s/seq-pq1JBIlQc241m0GCv8kl?m=slm-2o0PxNjAliqEN1vZ3jny	This study
2421	HB101 / pMTL-YN1C *spoIIE*	https://benchling.com/s/seq-wf12gO3wrIm2yiQF8yWy?m=slm-97F7LGCr0FknuisiDBmv	This study
610	BL21 (DE3) / pET-22b Spo0A-His6	pET-22b Spo0A (C-terminal His6) in BL21(DE3)	[2]
3359	BL21 (DE3) / pET-22b $Spo0A_{145\text{-}247}$-His6	https://benchling.com/s/seq-vVMEqbhz0o3wADrDMucj?m=slm-uJSH7w9wmhyRLtyfxPwU	This study, adapted from [4]
1860	BL21 (DE3) / pET-28a CamA-His6	https://benchling.com/s/seq-l8PkWb2sUW8JcL9HFdgx?m=slm-VMD9q62G4fgoTaEzWBM8	This study
3348	BL21 (DE3) / pET-22b $SpoIIE_{906\text{-}2370}$-His6	https://benchling.com/s/seq-9WKn2XLwVnd0qZGYcKIM?m=slm-RNteiTBI8mKTG1290Pkq	This study

1. Ng, Y.K. *et al.* Expanding the repertoire of gene tools for precise manipulation of the *Clostridium difficile* genome: allelic exchange using pyrE alleles. *PLoS One* **8**, e56051 (2013).
2. Putnam, E. E., Nock, A. M., Lawley, T. D. & Shen, A. SpoIVA and SipL Are Clostridium difficile Spore Morphogenetic Proteins. Journal of Bacteriology 195, 1214–1225 (2013).
3. Pereira, F. C. et al. The Spore Differentiation Pathway in the Enteric Pathogen Clostridium difficile. PLOS Genetics 9, e1003782 (2013)
4. Rosenbusch, K. E., Bakker, D., Kuijper, E. J. & Smits, W. K. C. difficile 630Δerm Spo0A Regulates Sporulation, but Does Not Contribute to Toxin Production, by Direct High-Affinity Binding to
