# Supplementary material for: A single DNA methylation site regulates cell fate during Clostridioides difficile sporulation
Source: PLoS Pathog. 2026 Jul 23;22(7):e1013845. doi: 10.1371/journal.ppat.1013845 (PMC13395437; doi:10.1371/journal.ppat.1013845)
Supplement: S4 Table — (PDF) [file ppat.1013845.s020.pdf]

**Supplementary Table 4.** *B. subtilis* strains used in this study.

| Lab Strain # | Strain Name | Relevant genotype or link to Benchling plasmid map with primers | Source/reference |
|---|---|---|---|
| 5024 | BDR123 | *B. subtilis* 178 *amyE::cat* | D. Rudner |
| 5032 | *B. subtilis* 178 *amyE::PspoIIE-mScarlet-I3-spec* | https://benchling.com/s/seq-l9kAgoI79a999BgsgHTt?m=slm-T3Cz4HzezRsCQclZjK3y | This Study |
