# Supplementary material for: A single DNA methylation site regulates cell fate during Clostridioides difficile sporulation
Source: PLoS Pathog. 2026 Jul 23;22(7):e1013845. doi: 10.1371/journal.ppat.1013845 (PMC13395437; doi:10.1371/journal.ppat.1013845)
Supplement: S5 Table — (PDF) [file ppat.1013845.s021.pdf]

Supplementary Table 5. Primers used in this study

Lab Primer #	Primer Name	Sequence	Source/reference
1002	5' *rpoB* (235-442) qPCR	GAGTGTAAAGAGAGAGATGC	[1]
1003	3' *rpoB* (235-442) qPCR	CTTCCGCATAGTAAACACC	[1]
1583	5' *spo0A* (1-442) qPCR	ATGGGGGGATTTTTAGTGG	[2]
1584	3' *spo0A* (1-442) qPCR	TCATTTGAGTCTCTTGAACTGGTC	[2]
1213	5' *spoIIQ* (190-553) qPCR	GATGCTATCCCTACTGCAACG	[1]
1214	3' *spoIIQ* (190-553) qPCR	GTCCTTCTGTTACCTTCTGTTC	[1]
3031	5' *spoIIE* (536) qPCR	GTACGGACAGAAGAAGCCATATC	[3]
3032	3' *spoIIE* (661) qPCR	TGGTAGCTCCACCTACTATTGA	[3]
4204	5' P*spoIIE* EMSA, IRD800-labeled	GTGATAAAAAGAACTTAAAAGGGCTACAAG	This study
4217	5' P*spoIIE* EMSA	GTGATAAAAAGAACTTAAAAGGGCTACAAG	This study
4205	3' P*spoIIE* EMSA	CCTCCTCTTTTGTTTATATTAAGAATTGTAAC	This study
4357	5' positive control FP, 6FAM-labeled	AATTTTGGAGTGTCGAATATGCTTTAGAGT	This study
4358	3' positive control FP	ACTCTAAAGCATATTCGACACTCCAAAATT	This study
4355	5' negative control FP, 6FAM-labeled	ATTTGATTTTTTAAAAAAATAAACA	This study
4356	3' negative control FP	TGTTTATTTTTTTAAAAAATCAAAT	This study
4359	5' WT P*spoIIE* FP, 6FAM-labeled	AAAACCGATTATGACAAAAAAAATTACTCC	This study
4360	3' WT P*spoIIE* FP	GGAGTAATTTTTTTTGTCATAATCGGTTTT	This study
4361	5' Me3* P*spoIIE* FP, 6FAM-labeled	AAAACCGATTATGACAAATAAAATTACTCC	This study
4362	3' Me3* P*spoIIE* FP	GGAGTAATTTTATTTGTCATAATCGGTTTT	This study
4319	5' WT PspoIIE MTase-Glo	AAGTCAAAAAAAATGTATTAATATTTTTTGATTTTTAAAACCGATTATGACAAAAAAAAT	This study
4320	3' WT PspoIIE MTase-Glo	ATTTTTTTTGTCATAATCGGTTTTAAAAATCAAAAAATATTAATACATTTTTTTTGACTT	This study

1. Fimlaid, K. A. et al. Global Analysis of the Sporulation Pathway of Clostridium difficile. PLoS Genet 9, e1003660 (2013).

2. Oliveira, P. H. et al. Epigenomic characterization of Clostridioides difficile finds a conserved DNA methyltransferase that mediates sporulation and pathogenesis. Nat Microbiol 5, 166–180 (2020).

3. Shrestha, S., Taib, N., Gribaldo, S. & Shen, A. Diversification of division mechanisms in endospore-forming bacteria revealed by analyses of peptidoglycan synthesis in Clostridioides difficile. Nat Commun 14, 7975 (2023).
